# Factors influencing Chongqing nurses' HPV vaccination: a cross-sectional survey based on the 5C model

**DOI:** 10.3389/fpubh.2026.1789086

**Published:** 2026-05-08

**Authors:** Mei Li, Qin Chen, Fulan Wang, Li Ding

**Affiliations:** 1 Department of Gynecology, The First Affiliated Hospital of Chongqing Medical University, Chongqing, China; 2 Department of Obstetrics and Gynecology, Chongqing Key Laboratory of Maternal and Fetal Medicine, Chongqing, China; 3 Joint International Research Laboratory of Reproduction and Development, Ministry of Education, Chongqing, China; 4 The Innovation and Talent Recruitment Base of Maternal-Fetal Medicine, The First Affiliated Hospital of Chongqing Medical University, Chongqing, China; 5 Department of Nursing, The First Affiliated Hospital of Chongqing Medical University, Chongqing, China

**Keywords:** 5C model, China, HPV vaccination, nurses, vaccine hesitancy

## Abstract

**Objective:**

This study aimed to investigate the key factors influencing HPV vaccination behavior among nurses in Chongqing, China, based on the 5C psychological antecedents model.

**Methods:**

A cross-sectional survey was conducted among 550 nurses from public hospitals in Chongqing between April 2024 and July 2024 via an online questionnaire. A combination of convenience and snowball sampling was employed. The instrument gathered demographic data, HPV vaccination status, and responses to the 5C scale (Confidence, Complacency, Constraints, Calculation, and Collective Responsibility).

**Results:**

The HPV vaccination rate among nurses was 52.7%. Hierarchical binary logistic regression analysis revealed that age (OR = 0.939, 95% CI: 0.895–0.985), influence from friends and colleagues (OR = 3.821, 95% CI: 2.634–5.542), and constraints (OR = 0.870, 95% CI: 0.811–0.933) were significant predictors of vaccination status. In contrast, confidence, complacency, calculation and collective responsibility were not statistically significant in the final model.

**Conclusion:**

The HPV vaccination behavior of nurses in Chongqing is significantly influenced by age, social influence and structural barriers. To improve vaccination coverage, interventions should focus on leveraging peer influence, fostering a supportive vaccination climate, and systematically reducing constraints through convenient access and financial support. These findings contribute to understanding vaccine hesitancy among healthcare workers and inform strategies to achieve cervical cancer elimination goals in China.

## Introduction

Approximately one fifth of new cervical cancer cases worldwide occur in China (1). The survey shows that the incidence of cervical cancer in China is continuously increasing and there is a significant trend of younger age at diagnosis (2). Human papillomavirus (HPV) infection is the most common sexually transmitted infection globally and a primary causative agent for various cancers, including cervical, anal, and oropharyngeal cancers, posing a significant public health burden (3). Given that HPV infection is the necessary cause of cervical cancer, vaccination represents the most effective primary prevention strategy. HPV vaccination is widely recognized as a cornerstone of primary prevention against HPV-related cancers (4), and its safety and efficacy have been strongly supported by extensive empirical evidence (5, 6). Many countries worldwide have incorporated HPV vaccination into their national immunization programs (7), achieving remarkable public health success. However, vaccination coverage, particularly among key populations, remains a major challenge in achieving the goal of cervical cancer elimination.

In recent years, with the approval of multiple HPV vaccines and increased public health awareness, vaccine accessibility has improved (8). From 2016 to 2018, China primarily imported HPV vaccines (including bivalent, quadrivalent, and nonavalent HPV vaccines) (9). The introduction of the domestic bivalent HPV vaccine in late 2019 further reduced vaccination costs (10), providing new opportunities to enhance vaccine coverage. However, during 2018–2020, cumulative full vaccination coverage among women aged 9–45 years was only 2.24% (11). By 2022, first-dose coverage among the same age group had risen to 10.15%, while full (three-dose) coverage remained low at 6.21% (12). This situation may be more pronounced in less developed or inland regions. As a major municipality in Western China with a large population and regional development disparities, understanding the vaccination behaviors of key groups in Chongqing is crucial. In China, HPV vaccination is currently recommended for women aged 9 to 45 (13), but it remains a voluntary, out-of-pocket service (mainly for adult women), and there is currently no national reimbursement policy in place (14). Some provinces have initiated pilot programs offering free or subsidized vaccination for adolescent girls (15).

Nurses, as a cornerstone of the healthcare system, serve not only as disseminators of vaccine knowledge but also as role models for health behaviors. Their vaccination behavior not only affects their personal health but also significantly influences public vaccination intentions (16). Research demonstrates that when healthcare workers are vaccinated themselves, they can recommend vaccines to patients more confidently, thereby improving overall vaccination rates (17). However, concerningly, although nurses generally possess high levels of knowledge about HPV and its vaccine, their own vaccination rates do not necessarily correspond. A large national cross-sectional study conducted in 2021 involving 15,967 female healthcare workers across 31 provinces reported that only 22.8% had received the HPV vaccine, with an additional 7.16% having made an appointment (18). This indicates a noticeable “knowledge-attitude-practice gap” (19). This gap may arise from multiple factors, including structural barriers (e.g., cost, time constraints), risk perception biases (e.g., complacency about personal risk), or other psychological antecedents that mediate the translation of knowledge into action (20).

Understanding vaccination behavior requires a robust theoretical framework. The 5C psychological antecedents model (21) is a validated framework that assesses an individual's vaccination intention through five core components: Confidence (trust in vaccine safety and effectiveness), Complacency (perceiving a low disease risk), Constraints (structural or psychological barriers), Calculation (extensive information searching and weighing), and Collective Responsibility (willingness to contribute to herd immunity). Compared to the general public, nurses typically possess higher levels of knowledge about HPV, yet their vaccination rates do not necessarily correspond. In this context, the 5C model is particularly relevant for studying nurses, as its ability to disentangle Confidence (trust in vaccines) from Complacency (low risk perception) and Constraints (structural barriers). This is especially important for analyzing populations with higher health literacy, as these factors are often conflated within simple knowledge frameworks. Investigating the 5C factors influencing their decisions thus moves beyond simple Knowledge-Attitude-Practice (KAP) surveys to uncover the complex underlying psychological drivers.

Currently, there is a scarcity of research on the HPV vaccination behavior among nurses in Western China based on well-established theoretical models. Therefore, This study aimed to investigate the key factors influencing HPV vaccination behavior among nurses in Chongqing, China, based on the 5C psychological antecedents model. Specifically, we sought to answer the following research questions: (1) What is the HPV vaccination rate among nurses in Chongqing? (2) Which 5C psychological antecedents (confidence, complacency, constraints, calculation, and collective responsibility) are independently associated with vaccination status? (3) Do demographic and professional characteristics predict vaccination after controlling for 5C factors? The findings will help fill this knowledge gap and provide empirical evidence for healthcare institutions and public health policymakers to design targeted interventions (e.g., reducing accessibility constraints) To increase the vaccination rate among the nursing staff.

## Materials and methods

### Study design and population

This was a cross-sectional study conducted among registered nurses working in clinical departments. The inclusion criteria were registered nurses currently practicing in clinical departments of the participating hospitals. Exclusion criteria: nurses who were on extended leave (including sick leave, maternity leave, or long-term personal leave) during the data collection period; nurses who had previously participated in the pilot test; nurses who were unable to provide informed consent due to cognitive impairment or other reasons.

Sample size was determined based on the events-per-variable (EPV) principle (22). Based on the EPV criterion of ≥10 events per predictor variable (22), with an expected vaccination rate of 50% (derived from the pilot data of 30/60) and approximately 18 potential predictors, a minimum of 360 participants was required. We plan to recruit more than 360 participants to ensure adequate statistical power. Given that it is difficult to establish a comprehensive sampling framework for all nurses in the Chongqing area, and considering the high work intensity of clinical nurses, we adopted the methods of convenience sampling and snowball sampling to recruit participants more efficiently. This method can quickly reach eligible nurses across different hospitals and departments in Chongqing, which is consistent with our research goal of investigating HPV vaccination factors among nurses in Chongqing.

### Measures

The questionnaire was structured based on the theoretical framework of the 5C model (Confidence, Complacency, Constraints, Calculation, and Collective Responsibility). It consisted of two main sections. Demographic and professional characteristics: This section collected information including age, gender, household registration (urban/rural), highest education level, sexual active, professional title, average monthly income, family history of cervical cancer and so on.

Vaccination Status and 5C Psychological Precursors: Vaccination status was measured using the question “Have you currently received the HPV vaccine?”. The 5C components were assessed using the 5C scale (21). The 5C Scale comprises 15 items organized into five factors: Trust, Complacency, Constraint, Calculation, and Collective Responsibility, each containing three items. There are 3 items in each dimension. Item 13 were reverse-coded. A 7-point scale ranging from 1 (strongly disagree) to 7 (strongly agree) was used. Higher scores on Trust and Collective Responsibility indicate lower vaccine hesitancy, while higher scores on Complacency, Constraints, and Calculation indicate higher vaccine hesitancy. Subscale scores were calculated by summing individual item scores (range: 3–21). The 5C scale was administered in its original English version, as the study sample consisted of nurses with advanced English proficiency and to ensure that the measurement reflects the original psychometric properties of the scale. A pilot test (*n* = 60) was conducted to assess item clarity, and Cronbach's α coefficients calculated from the full sample (*N* = 550) indicated acceptable internal consistency for all subscales (all coefficients > 0.7).

### Quality assurance

Several measures were implemented to ensure data quality: (1) Logical checks were embedded within the questionnaire; (2) Incomplete submissions (<95% completion) were excluded; (3) The minimum response time was set to 180 seconds to prevent random clicking; (4) Duplicate responses from the same IP address were removed.

### Data collection

Data were collected online via the widely used Chinese survey platform “Questionnaire Star” between April 2024 and July 2024. The survey link was created using the “Questionnaire Star” platform. We contacted nursing administrators at ten tertiary hospitals in Chongqing and asked them to share the link in their departmental WeChat groups. Recipients were also encouraged to forward the link to eligible colleagues.

Based on the total number of nurses employed in the participating hospitals' departments, we estimate that roughly 1,500 nurses received the invitation. A total of 566 responses were obtained, yielding a crude response rate of 37.7%. Because we could not track how many nurses actually opened the link or viewed the questionnaire, this figure should be interpreted as a rough approximation. In accordance with questionnaire quality requirements, 16 invalid questionnaires were ultimately excluded. There were 550 valid questionnaires for statistical analysis.

### Statistical analysis

Data analysis was performed using SPSS Statistics software (version 21.0, IBM Corp). Personal monthly income was originally collected in nine categories. Due to small sample sizes in some lower and higher income brackets, these were collapsed into four groups for analysis: <5,000, 5,001–10,000, 10,001–15,000, and >15,000. This categorization was based on approximate quartiles of the sample distribution to ensure adequate group sizes for statistical comparisons. Normality of continuous variables (age and the five 5C subscales) was assessed using the Kolmogorov–Smirnov test. Given that all continuous variables deviated significantly from normality (*p* < 0.05), they were described using medians and interquartile ranges (IQR). For non-normal continuous variables, the Mann-Whitney *U*-test is used to compare differences between the vaccinated and unvaccinated groups. Categorical variables were compared using the chi-square test (or Fisher's exact test when expected cell counts were <5). A two-tailed *p*-value of less than 0.05 was considered statistically significant.

To identify factors associated with HPV vaccination status, a hierarchical binary logistic regression model was constructed. Based on the theoretical framework of the 5C model, all five 5C subscales (Confidence, Complacency, Constraints, Calculation, and Collective Responsibility) were simultaneously entered into the model, together with demographic and professional variables that showed statistical significance in univariate analyses (age and influence by friends/colleagues). All variables were entered using the enter method (forced entry) to preserve the theoretical integrity of the model and to control for potential confounding among the psychological dimensions. To further examine whether demographic and professional characteristics contributed additional explanatory power beyond the 5C constructs, a hierarchical logistic regression approach was used: Model 1 included only the five 5C subscales; Model 2 added age and peer influence. Model improvement was assessed using changes in the −2 log-likelihood and Nagelkerke R^2^.

## Results

Based on the full study sample (*N* = 550), Cronbach's α coefficients were calculated for the total 5C scale and each of the five subscales. The total scale demonstrated acceptable internal consistency (α = 0.702). All five subscales showed good reliability, with Cronbach's α values ranging from 0.701 to 0.866: Confidence (α = 0.815), Complacency (α = 0.701), Constraints (α = 0.866), Calculation (α = 0.816), and Collective Responsibility (α = 0.826). These results indicate that the English version of the 5C scale exhibited satisfactory internal consistency in this sample of Chinese nurses. Multicollinearity was assessed using variance inflation factors (VIFs) calculated from a linear regression model with the same set of predictors. All VIFs were below 2.0, indicating that multicollinearity was not a concern in the final model.

A total of 550 nurses were included in the analysis. The median age of participants was 30 years (IQR: 27–33), and the majority were female (95.6%). Approximately half of the nurses (52.7%) had received the HPV vaccine. Detailed demographic and professional characteristics are presented in Table 1. Descriptive statistics for the five 5C subscales (Confidence, Complacency, Constraints, Calculation, and Collective Responsibility) are shown in Table 2. Scores for all subscales were non-normally distributed and are presented as medians with interquartile ranges (IQR). In the total sample, median scores ranged from 3 to 21 across subscales. When stratified by vaccination status, vaccinated nurses had slightly higher Confidence scores and lower Constraints scores compared to unvaccinated nurses.

**Table 1:** General characteristics of respondents (*n* = 550).

Variable	Groups	Frequency (*n*)	Frequency (*n*)
Age	–	30 (27, 33)	–
Gender	Female	526	95.6
	Male	24	4.4
Department	Gynecology	103	18.7
	Other	447	81.3
Education	Associate degree	62	11.3
	Bachelor's degree	447	81.3
	Master's degree and above	41	7.5
Household registration	Urban	335	60.9
	Rural	215	39.1
Sexual active	Have	479	87.1
	Not have	71	12.9
Title	Nurse	180	32.7
	Nurse practitioner	134	24.4
	Nurse–in–charge	214	38.9
	Associate senior nurse	22	4.0
Personal monthly income (yuan)	<5,000	141	25.6
	5,001–10,000	288	52.4
	10,001–15,000	110	20.0
	>15,000	11	2.0
Family history of cervical cancer	Have	8	1.5
	Not have	542	98.5
History of self-paid vaccination (non-HPV vaccines)	Have	267	48.5
	Not have	283	51.5
Do your family members support HPV vaccination?	Yes	525	95.5
	No	25	4.5
The decision on HPV vaccination is influenced by friends and colleagues?	Yes	264	48.0
	No	286	52.0
Have you been vaccinated against HPV?	Yes	290	52.7
	No	260	47.3

**Table 2:** Descriptive statistics of the 5C scale.

5C Subscale	Score range	Total sample (*N* = 550)	Unvaccinated (*n* = 260)	Vaccinated (*n* = 290)
Confidence	3–21	16 (13–19.25)	16 (13–19)	16.5 (14–20)
Complacency	3–21	3 (3–7)	4 (3–7)	3 (3–7)
Constraints	3–21	12 (10–14)	13 (11–15)	12 (9.75–14)
Calculation	3–21	12 (10–14)	12 (10–14)	12 (10–14)
Collective Responsibility	3–21	12 (10–14)	12 (10.25–14)	12 (10–14)

Univariate analyses comparing vaccinated and unvaccinated nurses are summarized in Table 3. Age, peer influence, and the Constraints subscale were significantly associated with vaccination status (all *p* < 0.05). No significant differences were observed for other demographic variables or the remaining 5C subscales. Hierarchical binary logistic regression analyses were performed to identify factors independently associated with HPV vaccination (Table 4). In Model 1, which included only the five 5C subscales, Constraints was the only significant predictor (OR = 0.834, 95% CI: 0.780–0.891, *p* < 0.001). In Model 2, which added age and peer influence, the Nagelkerke R^2^ increased from 0.079 to 0.207, indicating that the inclusion of age and peer influence variables substantially improved the model's explanatory power, with the explained variance increasing from 7.9% to 20.7%. Age (OR = 0.939, 95% CI: 0.895–0.985, *p* = 0.010) and peer influence (OR = 3.821, 95% CI: 2.634–5.542, *p* < 0.001) were significant predictors, while Constraints remained significant (OR = 0.870, 95% CI: 0.811–0.933, *p* < 0.001). Confidence, Complacency, Calculation, and Collective Responsibility were not significant in either model.

**Table 3:** Univariate analysis of demographic and 5C psychological factors.

Variable	Unvaccinated (*n* = 260)	Vaccinated (*n* = 290)	Test statistic	*p*-value
Age (years), median (IQR)	30 (28, 33)	30 (27, 32)	Z = −2.322	0.020
Gender			χ^2^ = 0.478	0.489
Female	247 (95.0)	279 (96.2)		
Male	13 (5.0)	11 (3.8)		
Department			χ^2^ = 3.309	0.069
Gynecology	57 (21.9)	46 (15.9)		
Other	203 (78.1)	244 (84.1)		
Education level			χ^2^ = 2.129	0.345
Associate degree	24 (9.2)	38 (13.1)		
Bachelor's degree	217 (83.5)	230 (79.3)		
Master's degree and above	19 (7.3)	22 (7.6)		
Household registration			χ^2^ = 0.057	0.811
Urban	157 (60.4)	178 (61.4)		
Rural	103 (39.6)	112 (38.6)		
Sexual active, *n* (%)			χ^2^ = 0.134	0.714
Have	35 (13.5)	36 (12.4)		
Not have	225 (86.5)	254 (87.6)		
Title			χ^2^ = 1.445	0.695
Nurse	83 (31.9)	97 (33.4)		
Nurse practitioner	69 (26.5)	65 (22.4)		
Nurse-in-charge	97 (37.3)	117 (40.3)		
Associate senior nurse	11 (4.2)	11 (3.8)		
Personal monthly income (RMB)			χ^2^ = 0.733	0.865
<5,000	63 (24.2)	78 (26.9)		
5,001–10,000	137 (52.7)	151 (52.1)		
10,001–15,000	55 (21.2)	55 (19.0)		
>15,000	5 (1.9)	6 (2.1)		
Family history of cervical cancer			–	1.000^*^
Have	4 (1.5)	4 (1.4)		
Not have	256 (98.5)	286 (98.6)		
History of self-paid vaccinations other than the HPV vaccine *n* (%)			χ^2^ = 3.049	0.081
Not have	144 (55.4)	139 (47.9)		
Have	116 (44.6)	151 (52.1)		
Family support HPV vaccination, *n* (%)			χ^2^ = 0.113	0.737
Yes	249 (95.8)	276 (95.2)		
No	11 (4.2)	14 (4.8)		
Influence by friends/colleagues, *n* (%)			χ^2^ = 64.009	<0.001
Yes	182 (70.0)	104 (35.9)		
No	78 (30.0)	186 (64.1)		
5C subscales (median, IQR)				
Confidence	16 (13–19)	16.5 (14–20)	Z = −1.111	0.267
Complacency	4 (3–7)	3 (3–7)	Z = −0.781	0.435
Constraints	13 (11–15)	12 (9.75–14)	Z = −5.020	<0.001
Calculation	12 (10–14)	12 (10–14)	Z = −0.015	0.988
Collective Responsibility	12 (10.25–14)	12 (10–14)	Z = −0.933	0.351

^*^: Fisher's exact test. Continuous variables (age and 5C subscales) were non-normally distributed (Kolmogorov-Smirnov test, *p* < 0.05) and are presented as median (interquartile range). Comparisons were performed using the Mann-Whitney *U*-test. Categorical variables were compared using the chi-square test or Fisher's exact test.

**Table 4:** Hierarchical multivariate binary logistic regression analysis of factors associated with HPV vaccination among nurses.

Variable	Model 1 (5C subscales only)		Model 2 (5C subscales + demographic/professional)	
	OR (95% CI)	*p*-value	OR (95% CI)	*p*-value
5C subscales				
Confidence	1.026 (0.978–1.075)	0.298	1.021 (0.971–1.074)	0.418
Complacency	0.981 (0.933–1.032)	0.467	0.970 (0.919–1.023)	0.261
Constraints	0.834 (0.780–0.891)	<0.001	0.870 (0.811–0.933)	<0.001
Calculation	1.079 (0.997–1.167)	0.059	1.082 (0.995–1.176)	0.065
Collective responsibility	0.998 (0.921–1.081)	0.955	0.984 (0.904–1.071)	0.705
Demographic/professional				
Age	—	—	0.939 (0.895–0.985)	0.010
Influence by friends/colleagues (Yes vs. No)	—	—	3.821 (2.634–5.542)	<0.001
Model fit				
−2 Log likelihood	727.318		668.130	
Nagelkerke R^2^	0.079		0.207	
Δ Nagelkerke R^2^			+0.128	

Model 1 included only the five 5C subscales. Model 2 added age and peer influence. All variables were entered using the enter method. OR, odds ratio; CI, confidence interval. All continuous variables (age, 5C subscales) were entered as continuous scores. The reference category for peer influence is “No.”

## Discussion

The HPV vaccination rate observed in our sample (52.7%) is lower than the 70.77% reported among female healthcare workers at HPV vaccination clinics in Beijing (23). Notably, adding age and peer influence variables to our model substantially improved its explanatory power, with the explained variance increasing from 7.9% to 20.7%. Our finding that constraints were a significant barrier aligns with previous research. A Beijing study (23) found that perception of high vaccine price was a significant negative predictor of HPV vaccination (OR = 0.616, 95% CI: 0.427–0.888), consistent with our identification of structural barriers as major obstacles. A systematic review of healthcare workers' HPV vaccine recommendation practices further identified cost, time constraints, and systemic barriers as the main obstacles to vaccine recommendation (24). Regarding the 5C model, a Shanghai study of 1,928 nurses applied the framework and identified four vaccine hesitancy profiles, with “believers” (high confidence and collective responsibility) comprising 68.9% of the sample (25). Our study thus contributes novel evidence on the differential predictive value of 5C dimensions specifically for HPV vaccination in a Chinese nursing population, showing that structural barriers (Constraints) and social influences (peer norms) outweigh attitudinal factors such as Confidence and Complacency in predicting vaccination behavior.

Our findings indicate that the likelihood of HPV vaccination decreased with increasing age. To interpret this age gradient, it is important to consider the policy context of HPV vaccination in China during the period when participants were making vaccination decisions. The imported bivalent vaccine, quadrivalent vaccine, and nine-valent vaccine were respectively, approved in 2016, 2017, and 2018 (26). The first domestic bivalent vaccine became available in late 2019 (27), substantially improving affordability and supply. Throughout the time of our data collection (April–July 2024), HPV vaccination remained a self-paid, voluntary service. Against this backdrop, the age effect likely reflects a cohort effect: younger nurses entered early adulthood when domestic vaccines became widely available and were thus more likely to perceive themselves as eligible and at risk; they are also more accustomed to obtaining health information via digital media and show higher acceptance of novel preventive measures (28). These findings carry important implications for intervention. Health education for nurses should be age-specific and emphasize that HPV vaccination provides benefits even for sexually experienced individuals, including protection against non-acquired HPV types and prevention of progression from infection to precancerous lesions.

Our analysis reveals that having one's vaccination decision influenced by friends and colleagues was one of the strongest positive predictors of HPV vaccine uptake (OR = 3.821, *p* < 0.001). This finding strongly aligns with social cognitive theory and social norms theory (29). This peer effect not only conveys a descriptive norm (i.e., “most people like me are getting vaccinated”) but may also provide practical advice on managing side effects or scheduling vaccinations through informal daily communication, thereby reducing perceived behavioral barriers. However, it is important to note that this variable is self-reported and may be subject to social desirability bias or reverse causation—vaccinated individuals may be more likely to perceive influence from their peers. In practice, peer-led education sessions, testimonials from vaccinated nurses, and visible endorsement by respected colleagues could effectively harness this effect. Additionally, the influence of peers may vary by age or department; for example, younger nurses may be more susceptible to peer influence than their older counterparts.

This study confirms that perceived constraints are a key barrier to HPV vaccination among nurses (OR = 0.870, *p* < 0.001). These constraints primarily include the financial burden of high out-of-pocket costs, time conflicts between heavy clinical workloads and vaccination appointments (“time poverty”), and inconvenient transportation to vaccination sites (19, 20). In China, HPV vaccination is not universally covered by public insurance for adults. The domestic bivalent vaccine costs approximately 300–350 RMB per dose (three doses total), while imported vaccines range from 600–1,300 RMB per dose (29). With the median monthly income of nurses in our sample falling into the 5,001–10,000 RMB category (Table 1), the full course of vaccination represents a substantial financial outlay, particularly for younger nurses who may have lower incomes and greater competing expenses. Constraints may be particularly acute for frontline clinical staff with irregular shift patterns, who may struggle to schedule appointments during standard clinic hours. This aligns with studies identifying organizational and structural barriers to HPV vaccination elsewhere (30). Healthcare institutions should consider implementing targeted measures, such as establishing convenient on-site employee vaccination clinics, providing subsidies or group purchase discounts, and allowing vaccination during work hours, to systematically reduce these external barriers, thereby translating the high awareness and positive intentions of nurses into protective vaccination behavior.

Notably, confidence and complacency did not emerge as significant predictors of HPV vaccination uptake in the final multivariate model. One plausible explanation is a ceiling effect: as frontline healthcare professionals, nurses generally maintain high baseline confidence in vaccine safety and efficacy (31), leading to limited variability in these psychological scores that weakens their predictive power. Indeed, in our sample, Confidence scores were uniformly high (median 16, IQR 13–19.25) with little difference between vaccinated and unvaccinated groups (*p* = 0.267), supporting a ceiling effect. Alternatively, the non-significance may reflect the knowledge-attitude-practice gap—a well-described phenomenon in which high knowledge and positive attitudes do not automatically translate into behavior when practical barriers exist (32). In this context, even nurses with high confidence may fail to vaccinate if faced with structural hurdles such as cost or time constraints. Moreover, structural barriers (e.g., time constraints) and social influences (e.g., peer norms) may outweigh individual cognitive factors in driving vaccination behavior among this population. In our final model, Constraints and peer influence were the dominant predictors, suggesting that when practical obstacles are present and salient, they may override attitudinal factors. This indicates that interventions aimed at increasing HPV vaccination among nurses should prioritize reducing practical constraints and leveraging social influence rather than focusing primarily on boosting confidence or reducing complacency.

### Strengths and limitations

This study has several strengths. First, it is one of the first to apply the validated 5C psychological antecedents model to examine HPV vaccination behavior among nurses in Western China, providing theory-driven insights. Second, the hierarchical logistic regression approach allowed us to distinguish the unique contributions of psychological, demographic, and social factors. Third, the identification of modifiable factors (peer influence and structural constraints) offers concrete targets for intervention. However, several limitations should be considered in our findings. First, the cross-sectional design precludes causal inferences. Second, the convenience and snowball sampling method via WeChat may have introduced selection bias, likely overrepresenting nurses who are vaccinated, hold strong opinions about HPV vaccination, or have greater digital access. Therefore, this limits the generalizability of the research results to a broader nursing population in Chongqing and other regions of China. Third, all data were self-reported, which could be subject to recall bias and social desirability bias. Fourth, the study was conducted exclusively at tertiary hospitals in Chongqing, restricting the applicability of findings to other regions with different contexts. Fifth, despite adjusting for multiple covariates, residual confounding from unmeasured factors (e.g., prior HPV infection, institutional policies, parity or menopausal status) cannot be ruled out. Additionally, due to sample size considerations, we did not examine potential interaction effects (e.g., between peer influence and age or constraints); future studies with larger samples should investigate these moderating relationships. Sixth, the use of the original English version of the 5C scale, while appropriate for our sample's proficiency, lacks formal validation in Chinese, which may affect cultural and semantic equivalence. No formal translation or back-translation was performed, as all participants demonstrated sufficient English proficiency as part of their professional qualifications.

## Conclusions

This study confirms that the 5C psychological antecedents model provide a valuable framework for identifying the complex factors shaping HPV vaccination decisions among nurses in Chongqing, with peer influence and constraints emerging as the dominant predictors. The findings demonstrate that younger age (each additional year of age reduced the odds of vaccination by approximately 6%), positive peer influence (which nearly quadrupled the odds of vaccination), and fewer perceived constraints significantly increase the likelihood of vaccination. This underscores that vaccination decisions, even among highly knowledgeable healthcare professionals, are not solely based on rational cognition but are co-shaped by social norms, practical barriers, and personal risk perception. Consequently, health education alone is insufficient to substantially improve uptake. Future intervention strategies must be multi-pronged: cultivating a supportive environment within healthcare institutions by harnessing the demonstrative effect of “vaccine champions,” while healthcare administrators must proactively address structural constraints through tangible support such as on-site vaccination services, subsidies or group purchasing to reduce costs, paid time off for vaccination, and peer-led education campaigns featuring vaccinated nurse champions. By targeting these key modifiable factors, we can effectively bridge the knowledge-attitude-practice gap, enhance HPV vaccine coverage among nurses and the communities they serve, and contribute to China's efforts to eliminate cervical cancer.

## Data Availability

The raw data supporting the conclusions of this article will be made available by the authors, without undue reservation.
